# Septicæmia

**Published:** 1905-03-18

**Authors:** 


### SEPTICÆMIA.

The march of bacteriology is constantly shedding fresh light on dark places, and though the technique of the study often puts it beyond the pale of routine use, the results already obtained contain many points important both as to practice and as influencing our conceptions of the rôle played by certain of the pathogenic micro-organisms. An admirable summary appears in a communication by Dr. T. J. Horder,[1] in which he details the fruits of 14 months' experience in this field at St. Bartholomew's Hospital. The method, briefly stated, consists in the withdrawal, under completely aseptic conditions, of 5 cc. of blood from any vein at the bend of the elbow, with which blood culture-tubes of sterile broth, or other media, are immediately inoculated. The tubes are then incubated at body temperature, and (if broth has been used) colonies of any organisms free in the blood-stream appear in the clot formed in the medium. By this means it has been found that a large variety of microbes are capable of giving rise to septicæmia (using this word to signify a condition in which pathogenic organisms are free and multiplying in the circulation).

Clinically the subjects of this condition present obscure symptoms roughly divisible into two classes: (*a*) Those presenting a low fever without local symptoms, a class frequently suspected of being typhoid fever; and (*b*) those presenting signs of endocarditis with low fever, cases grouped together as malignant endocarditis. The organisms which have from time to time been recovered from the blood by the method above detailed, or similar ones, are the streptococcus pyogenes, the staphylococci aureus and albus pyogenes, the pneumococcus (these four often), the bacilli of tuberculosis, influenza, typhoid fever, plague, anthrax, and glanders, and the cocci of gonorrhœa, rheumatism, and cerebrospinal meningitis.

These results must tend to moderate preconceptions in several points. In the case of gonorrhœa, for instance, we have most of us been taught to believe that the infecting organism levelled itself chiefly at the mucous membranes, the occurrence of gonorrhœal arthritis being a secondary toxic phenomenon. But now it has been on several occasions established that the gonococcus can and does invade the blood-stream, for it has been recovered from the vegetations of certain cases of malignant endocarditis. Dr. Horder mentions one such case in his series, and another, occurring in China, has just been reported by Dr. William Hunter.[2] Again, influenza has been known to be associated with endocarditis, but never before has it been proved to demonstration that the influenza bacillus was the actual exciting cause of the endocarditis by its presence in the blood-stream.

The practical bearing of these researches touches the matter of serum therapeutics. It has been too generally assumed that patients presenting the clinical picture of a septicæmia have been infected with the streptococcus pyogenes, and on this assumption anti-streptococcic serum has frequently been administered. But it is clearly futile to inject with a serum possibly protective against the streptococcus a patient whose illness is due to the influenza bacillus or any other of the numerous organisms mentioned above. Dr. Horder believes that the recovery of a pathogenic organism from the blood of any patient is of the gravest possible significance, and that the chief hope of remedy lies in the direction of specific serums. Unfortunately, happy as have been the effects of serum treatment in the case of organisms such as those of diphtheria, whose poisons only enter the circulation, little progress has so far been made in the fight, on similar lines, with those able themselves to enter and multiply in the blood-stream.

[1] St. Bartholomew's Hospital Journal, March 1905.
[2] Brit. Med. Jour., March 11, 1905.

